# The Role of Myocardial Strain Imaging in the Pre- and Post-Operative Assessment of Patients with Single Ventricle

**DOI:** 10.31083/j.rcm2405145

**Published:** 2023-05-18

**Authors:** Panagiota Kleitsioti, George Koulaouzidis, Pinelopi Giannakopoulou, Dafni Charisopoulou

**Affiliations:** ^1^Cardiology Department, G Papanicolaou Hospital, 57010 Thessaloniki, Greece; ^2^Department of Biochemical Sciences, Pomeranian Medical University, 70-204 Szczecin, Poland; ^3^Paediatric Cardiology Department, Amalia Children’s Hospital, Radboud University Medical Centre, 6525 GA Nijmegen, Netherlands; ^4^Paediatric Cardiology Department, Academic Centre for Congenital Heart Disease, 6500 HB Nijmegen, Netherlands

**Keywords:** hypoplastic left heart syndrome, single ventricle, speckle-tracking echocardiography, strain, strain rate, outcomes

## Abstract

The term “single ventricle” refers to a wide range of cardiac structural and functional abnormalities which cause the morphologically right or left ventricle to be hypoplastic or functionally inadequate. Patients with single-ventricle physiology have followed a series of palliative surgeries, resulting in the dominant ventricle supporting only the systemic circulation and the systemic venous return draining directly to the pulmonary arteries. Such patients present a progressive decline in myocardial performance, and their management is associated with high morbidity, mortality and resource usage. At each management step, imaging is critical in eligibility assessment, pre-procedural planning and prompt detection of myocardial dysfunction. However, the complex and asymmetric geometry of the dominant ventricle and its segmental wall motion abnormalities make the echocardiographic evaluation of myocardial performance in these patients rather challenging. Consequently, conventional 2-dimensional echo functional parameters, such as ejection fraction by Simpson’s biplane method or shortening fraction by M-mode, is complex and often not feasible to apply. On the other hand, speckle-tracking echocardiography is angle and geometry independent and has better reproducibility. As such, it constitutes an appealing method for assessing myocardial function in patients with single-ventricle hearts. Therefore, this review aims to investigate the role of myocardial strain imaging by speckle-tracking echocardiography in the pre-and post-operative assessment of patients with single-ventricle hearts.

## 1. Introduction

Patients with single-ventricle (SV) physiology are among the highest-risk congenital heart disease patients with increased mortality and morbidity, frequently caused by ventricular dysfunction [1]. These patients undergo staged surgical management with the end result of the Fontan or total cavopulmonary circulation, where the dominant ventricle supports only the systemic circulation. In contrast, systemic venous return drains directly into the pulmonary circulation without mixing with the pulmonary venous return [2]. Depending on the underlying type of pulmonary and systemic blood flow, most newborns with single-ventricle will require intervention to secure systemic blood flow and control pulmonary circulation. This is commonly achieved with the Norwood stage 1 procedure [2]. This will be followed by superior bidirectional cavopulmonary anastomosis (BCPA) or Norwood stage II, performed at 4 to 6 months of age, and a Fontan completion (stage III) at 18 to 48 months of age [2]. Patients with single ventricle physiology present a progressive decline in myocardial performance due to combined structural and functional disturbances [3]. Abnormal direction of myofibers in the dominant ventricle, increased myocardial fibrosis, abnormal ventricular outflow morphology and non-contractile septum were shown to be among the potential contributing factors [3].

Cardiac magnetic resonance imaging (MRI) is the ‘gold’ standard for non-invasively assessing ventricular volume and function. As such, it is an additional tool for evaluating single ventricle patients, especially when deciding Fontan eligibility [4]. However, its use for frequent serial assessments presents limitations, including lack of availability in all centers, the need for sedation in young children, lengthy analysis times and higher costs [4]. On the other hand, transthoracic echocardiography can be used on children without sedation, is readily available, more cost-effective, faster and easier [4]. As such, it can be used for the routine serial assessment of these patients. However, the complex asymmetric geometry of the dominant ventricle and the potential presence of segmental wall motion abnormalities make the echocardiographic evaluation of myocardial performance in single-ventricle patients challenging [5]. Therefore, conventional 2-dimensional (2D) echo measurements of ventricle function, such as ejection fraction either by Simpson’s biplane method or by M-mode shortening fraction, cannot be used as in normal biventricular hearts [6]. Due to its angle- and geometry independence and good reproducibility, speckle-tracking echocardiography (STE) may have significant potential for assessing single-ventricle patients [7, 8, 9]. Moreover, STE indices are more sensitive than conventional echocardiography parameters, identifying early ventricular function changes [10, 11, 12].

This review aims to examine the role of speckle-tracking echocardiography in the pre-and post-operative assessment of single-ventricle patients and to explore its prognostic value.

## 2. Ventricular Function Assessment by STE

During the last decade, several studies used STE to assess global and regional ventricular deformation and mechanical synchrony in patients with SV physiology, to understand the progress of intrinsic myocardial function during the various palliation surgical stages and to identify prognostic markers. However, most of these studies are retrospective and included patients with hypoplastic left heart syndrome (HLHS), whereas only a few included patients with other anatomic types of the single ventricle.

### 2.1 At Baseline, before the Norwood Operation

Before the first-stage palliative intervention in neonates with HLHS, the dominant right ventricle (RV) exhibits lower strain and strain rate parameters compared to the right and left ventricles (LV) of normal biventricular hearts. Altit *et al*. [13] conducted a study comparing conventional and speckle-tracking echocardiography indices in 34 newborns with HLHS and 28 controls (Table 1) They found that although there were no differences in RV fractional area change (FAC) between the two groups, RV tricuspid annulus peak systolic excursion (TAPSE) and RV systolic longitudinal strain and strain rate were significantly lower in the HLHS group. Moreover, the dominant RV longitudinal and circumferential strain and strain rate in HLHS patients were lower than the corresponding parameters of the LV in newborns with normal biventricular hearts (*p *< 0.0001).

**Table 1.** **Studies assessed the function of the single ventricle**.

Study	Country	Centers	Type of study	SV	Controls	Heart disease	Operation	Aim	Follow-up period	Echocardiographic parameters
Campbell [23] 2020	USA	single center	retrospective	40	n/a	Dominant RV (22)	Fontan (LT & EC), fenestration	ventricular function in patients with SV after Fontan operation	10.1 (9.1–10.6) years	GLS & GLSR, FWS & FWSR, GCS & GCSR, S/D ratio
Altit [13] 2019	USA	single center	retrospective	34	28	HLHS	n/a	RV performance in HLHS vs. controls postnatally	589 ± 428 days	GCS, GLS, GRS, GLSR, EDSR
Forsha [19] 2020	USA	single center	retrospective	20	n/a	HLHS	none, Norwood	effect of varying LV size on regional RV mechanics	n/a	RV GLS
Petko [25] 2011	Germany	single center	retrospective	29	n/a	HLHS	Norwood (modified BT shunt), CAPA, Fontan	RV deformation and dyssynchrony in HLHS with different anatomical subtypes after Fontan surgery	n/a	peak regional SLS & SLSR, GLS & GLSR, dyssynchrony, LVEDA, RVF
Markkanen [16] 2013	Finland	single center	retrospective	66	n/a	HLHS	Norwood	impact of prenatal diagnosis of HLHS on postnatal myocardial function	n/a	RV FAC, global & segmental myocardial velocities, GSR, SSR, mechanical synchrony
Menon [18] 2011	USA	single center	retrospective	30	n/a	HLHS	Norwood (RV-PA)	global and regional RV wall motion abnormalities after Norwood palliation	n/a	RV peak & average systolic circumferential & radial velocity, strain & SR, GCMDi, GLMDi
Michel 2016 [22]	Germany, USA	single center	retrospective	40	n/a	HLHS	TCPC	systolic and diastolic RV function within a 5-year follow-up period of HLHS patients after TCPC	5 years	RVFAC, TAPSE, E/A, E/e’, GLS, GLSR, GSRe, GSRa
Miller [14] 2012	USA	single center	retrospective	30	30	HLHS	n/a	regional mechanics of the fetal RV in HLHS vs. controls	n/a	velocity, GLS & SR, regional strain &SR
Ruotsalainen [17] 2017	Finland	single center	retrospective	63	-	HLHS	Blalock-Taussig, RV-PA	RV systolic performance/ volume load through the 3 surgical stage palliation	6–32 months	FAC, strain, SR, mechanical synchrony
Stiver [26] 2015	USA	single center	prospective	35	25	HLHS	Hybrid procedure/Norwood (BT)	diastolic dyssynchrony in patients with HLHS vs. control	3 ± 1.3 years	RV FAC, TDI S’ & e’, QRSe’ (LV, RV, IVS), Sre
Tham [20] 2014	Canada	single center	prospective/cross-sectional	76	30	HLHS	pre-Norwood, pre-BCPA, pre-Fontan, post-Fontan	trends in single RV systolic function between staged palliative surgeries	4 ± 9 months	TAPSE, S velocity, RV EDA, FAC, GLS & SR, GCS & SR, PSSi, mechanical dyssynchrony
Zaidi [27] 2019	USA	single center	retrospective	41	-	HLHS	Sano shunt	relationship between RV mechanical dyssynchrony, RV systolic function, and QRS duration	2 years	FAC, RV GLS, tPS-9, RVDi

BCPA, bidirectional cavopulmonary anastomosis; BT, Blalock-Taussig; CAPA, cavo-atriopulmonary anastomosis; EC, extracardiac conduit; EDA, end diastolic area; FAC, fractional area change; FWS, free wall strain; FWSR, free wall strain rate; GCS, global circumferential strain; GCSR, global circumferential strain rate; GLMDi, global longitudinal myocardial deformation index; GLS, global longitudinal strain; GLSR, global longitudinal strain rate; GRS, global radial strain; GCMDi, global circumferential myocardial deformation index; GSRa, global strain rate in late diastole; GSRe, global strain rate in early diastole; HLHS, hypoplastic left heart syndrome; IVS, interventricular septum; LT, lateral tunnel; LV, left ventricle; PSSi, post systolic strain index; QRSe’, The time interval measured from the QRS complex on ECG onset to the peak of the e’ wave; RV, right ventricle; RVDi, RV dyssynchrony index; RVF, right ventricular function; SD, standard deviation; SDe, septal defect; SLS, segmental longitudinal strain; SLSR, segmental longitudinal strain rate; Sre, The time interval measured from the QRS onset to the peak strain rate early diastolic wave; SV, single ventricle; TA, tricuspid atresia; tPS-9, SD of the time to peak strain for 9 segments; RVFAC, right ventricle fractional area change; TAPSE, tricuspid annulus peak systolic excursion; RV-PA, right ventricle to pulmonary artery; SR, strain rate; TCPC, total cavopulmonary connection; SSR, systolic strain rate; LVEDA, left ventricular end diastolic area; GSR, global strain rate; E, early ventricular filling velocity; A, late ventricular filling velocities; e’, early diastolic mitral annular velocity; ECG, Electrocardiogram; TDI S’ & e’, tissue doppler imaging systolic wave & early diastolic wave .

These strain and strain rate abnormalities in HLHS patients appear to be present prenatally. Miller *et al*. [14] compared 30 fetal patients with HLHS to 30 fetuses with normal hearts and found that global RV systolic strain was significantly lower in the fetuses with a single right ventricle (Table 1). Furthermore, a prenatal diagnosis of HLHS has been shown to improve postnatal outcomes [15]. Indeed, newborns with a prenatal diagnosis of HLHS have better single RV function, as assessed by strain and strain rate (Table 1) [16].

### 2.2 The Effect of Stage 1 Palliation and the Shunt Type on Myocardial Function 

Ruotsalainen *et al*. [17] conducted a study using velocity vector imaging (VVI) to examine the impact of shunt type [Blalock-Taussig (BT) shunt vs right ventricle to pulmonary artery (RV-PA) conduit] on myocardial strain and strain rate in 23 hypoplastic left heart (HLH) patients who received BT shunt and 40 HLH patients with RV-PA conduit (Table 1). Before the stage 1 surgical palliation, there were no differences in right ventricle fractional area change (RVFAC) or strain and strain rate parameters between the two groups. However, before stage 2 surgical palliation (BCPA), FAC was better in patients with RV-PA conduit. After stage 3, the BT shunt group showed better RV systolic function, as assessed by FAC, peak systolic longitudinal strain, and strain rate. Multiple regression analysis revealed that shunt type and the stage of palliation were the two factors impacting myocardial function.

Menon *et al*. [18] also investigated the effect of RV-PA conduit on ventricular function using VVI in 30 patients with HLHS (Table 1). While there were no differences in peak systolic strain and strain rate among the segments of the free wall and the septum of the RV, these parameters were significantly lower at the apex than at the base and mid-ventricle. These wall motion abnormalities were attributed to the ventriculotomy performed for the RV-PA conduit and persisted after stage 2 palliation. Long-term studies utilizing the easier applied STE could help evaluate the effects of ventriculotomy scar on a single systemic right ventricle.

### 2.3 The Effect of LV Size on RV Function 

Regional RV function assessment with STE was employed by Forsha *et al*. [19] in their study of 20 HLHS patients. Patients were divided into two groups according to the left ventricle size: diminutive/no visible LV (group 1) and moderate but unable to support the systemic circulation LV (group 2). Before stage-1 operation, the two groups had no differences in the dominant right ventricular (RV) global strain. However, patients with moderate but inadequate LV (group 2) presented significantly lower RV basal septal peak strain. In these patients, the septal strain was also considerably lower than RV apical and lateral wall strain, a relationship which was not observed in patients with diminutive LV (group 1). This RV segmental strain heterogeneity was preserved in the pre-stage-2 operation phase in group 2 patients. It was still not seen in group 1, despite no differences between them in global or segmental RV strain at that stage.

Group 2 patients also had worse clinical outcomes (death/transplant need). The authors suggested that HLH anatomic types with more significant LV or septal size had a greater degree of asymmetric RV mechanics which may be associated with worse outcomes. However, they have acknowledged the limitations derived from their small cohort sample.

### 2.4 Progression from Baseline to Fontan Completion 

In patients with HLH, in whom a decline in RV function is associated with a worse long-term prognosis, speckle-tracking echocardiography may complement conventional echocardiography in serially assessing RV function between staged surgeries and after Fontan completion. Tham *et al*. [20] evaluated conventional and STE-derived strain and strain rate parameters, such as post-systolic strain index (PSSi: peak strain divided by peak systolic strain) and mechanical dyssynchrony (standard deviation of segmental time to peak strain), in 76 HLHS patients at four stages of palliation (pre-Norwood, pre-BCPA, pre-Fontan, and post-Fontan) compared to 30 healthy individuals (Table 1).

While RV fractional area change (FAC) and myocardial performance index remained stable among conventional echocardiography parameters assessing RV function across the four stages, indexed TAPSE appeared to decrease from baseline (pre-Norwood) to pre- and post-Fontan phases. Similarly, longitudinal and circumferential RV strain rate was highest in the pre-Norwood period and decreased during the inter-stage phases, whereas it remained stable in controls. Furthermore, longitudinal RV strain was highest in the pre-Norwood period and subsequently decreased, whereas in controls, it increased. However, circumferential RV strain remained stable in all stages. As a result, HLHS patients exhibited a progressive decrease in the longitudinal to circumferential strain ratio during the palliation stages. Moreover, longitudinal post-systolic strain index (PSSi) was more significant at the pre-BCPA stage than at other stages, and circumferential PSSi was significantly more important at the pre-BCPA and pre-Fontan stages. Lastly, there was no difference in mechanical dyssynchrony across the four stages.

### 2.5 Eligibility for Fontan Completion 

The potential role of STE in deciding eligibility for Fontan completion was examined by Pasqua *et al*. [21]. This study included 21 patients with single left or right ventricles deemed eligible for proceeding to Fontan completion. The results showed that non-eligible patients exhibited significantly lower peak systolic longitudinal strain than the eligible ones. Moreover, lower peak systolic longitudinal strain was associated with higher end-diastolic left ventricle pressure and higher mean pulmonary pressure

### 2.6 Progression after Fontan Completion

Michel *et al*. [22] utilized conventional and speckle-tracking echocardiography to evaluate changes in systolic and diastolic right ventricular performance in HLHS patients after Fontan completion. During the follow-up period (1.6 to 5.1 years following total cavopulmonary connection [TCPC]), RVFAC, E/A, and E/e’ (E, early ventricular filling velocity; A, late ventricular filling velocities; e’, early diastolic mitral annular velocity) remained unchanged, while TAPSE decreased significantly. Additionally, both systolic and early and late diastolic RV strain rate values decreased considerably, despite no changes noted in global RV strain. These findings suggest that strain rate indices may aid in identifying early RV dysfunction.

Similarly, Campbell *et al*. [23] demonstrated in their study of 40 patients with a single right or left ventricle that global longitudinal and circumferential strain rate decreased over 10 years after the Fontan operation, despite no significant changes in conventional echocardiography parameters of ventricular function (Table 1). Furthermore, there were no significant differences noted in ventricular function during follow-up between patients with a single left or right ventricle or between those who met the endpoint of death or heart transplant (HT) and those who did not.

In a prospective study, Grattan *et al*. [24] revealed that patients with left single ventricles exhibited significantly better negative longitudinal and circumferential strain than patients with single right ventricles in the pre- or post-Fontan completion period. However, the latter patients had substantially greater end-diastolic volumes, as assessed by MRI (Table 1) [24]. Additionally, in this study, global ventricular torsion remained preserved in patients with right or left single ventricle pre- and post-Fontan completion, attributed to the increased apical torsion noted in single-ventricle patients (n = 61) compared to normal controls (n = 30).

Furthermore, Petko *et al*. [25] demonstrated that early after Fontan completion, HLH patients with mitral and aortic atresia presented distinct differences in regional deformation parameters and intraventricular dyssynchrony compared to other anatomical subtypes of HLHS (Table 1). However, no differences were noted between global longitudinal strain (GLS) and strain rate (SR).

Lastly, Stiver *et al*. [26] examined diastolic dyssynchrony in 35 HLHS children who had undergone Fontan completion and compared them with 25 children with normal hearts (Table 1). The time interval from the onset of QRS to the peak early diastolic strain rate (SRe) was obtained for the six RV segments. The standard deviation for the six SRe time intervals was calculated and used to indicate RV diastolic dyssynchrony. HLHS patients had significantly more pronounced RV diastolic dyssynchrony compared to controls. However, the degree of RV dyssynchrony did not seem to differ between patients who developed adverse events and those who did not. Nevertheless, in their study of 41 HLHS patients at various stages of palliation, Zaidi *et al*. [27] demonstrated that RV diastolic dyssynchrony was worse in patients with impaired single ventricle function, as assessed by FAC and GLS, suggesting a potential beneficial role of resynchronization therapy (Table 1).

## 3. Prognostic Role of STE Ventricular Function Assessment 

Multiple studies have investigated the potential use of ventricular strain and strain rate as prognostic indicators in patients with single ventricle physiology. Colquitt *et al*. [28] conducted a prospective study on 35 infants with HLHS, which revealed that post-Norwood, pre-Glenn, and post-Glenn STE parameters could predict adverse outcomes, including death, heart transplant, and persistent or moderate RV dysfunction (Table 2). Reduced longitudinal and circumferential strain and strain rate indices were identified as markers of adverse outcomes, with post-Norwood global longitudinal strain (GLS) >–16% and pre-Glenn GLS >–13% showing the highest sensitivity and specificity.

**Table 2.** **Studies investigate the prognosis of patients with single ventricle**.

Study	Country	Number of centers	Type of study	No of subjects	No of controls	Heart disease	Operation	Follow-up period	Aim	Echo parameters
Altit [13] 2019	USA	single center	retrospective	34	28	HLHS	n/a	589 ± 428 days	association of markers at the first echocardiogram with later mortality or HT	GLS, GLSR, RV FAC, RV TAPSE, Circumferential strain & SR
Borrelli [30] 2020	UK	single center	retrospective	27	n/a	HLHS	BCPA	1.18 ± 1.16 years	ability of serial STE and 2D echocardiography of RV in predicting death or HT	LS, LSR, TAPSE, FAC
Lin [29] 2018	Canada & USA	two centers	prospective	64	n/a	HLHS	BCPA	5.0 (2.8–6.4) years	diagnostic performance of STE and 2D echocardiography of RV in predicting death or HT	LS, LSR, CS, CSR, TAPSE, FAC
Park [31] 2017	USA	single center	retrospective	135	n/a	LV-dominant RV-dominant	TCPC	9–14 days	association between STE measurements & LOS	LS, LSR, CS, CSR, TAPSE, FAC
Colquitt [28] 2019	USA	single center	retrospective	35	n/a	HLHS	Norwood, BCPA	10.9 (5.6–15.2) months	STE of RV function is associated with poor cardiac outcome	LS, LSR, CS, CSR, TAPSE, FAC
Ghelani [32] 2015	USA	single center	retrospective	127	n/a	LV-dominant RV-dominant	Fontan	3.8 (2.6–5.7) years	association of STE and CMR parameters with death or HT	LS, LSR, CS, CSR

HLHS, hypoplastic left heart syndrome; BCPA, bidirectional cavopulmonary anastomosis; RV, right ventricle; LV, left ventricle; STE, speckle-tracking echocardiography; 2D, two-dimensions; HT, heart transplantation; PA, TAPSE, tricuspid annulus peak systolic excursion; FAC, fractional area change; LS, longitudinal strain; LSR, longitudinal strain rate; GLS, global longitudinal strain; GLSR, global longitudinal strain rate; SR, strain rate; CMR, cardiac magnetic resonance; CS, circumferential strain; CSR, circumferential strain rate; LOS, length of stay.

Lin *et al*. [29] examined the sensitivity and specificity of conventional and speckle-tracking echocardiography measures of RV function in predicting death or need for heart transplantation in a group of 64 HLHS patients before bidirectional cavopulmonary anastomosis (Table 2). The study found that patients with the endpoint had lower longitudinal strain and strain rate, circumferential strain rate, and RVFAC when compared to the rest of the patients. While both STE indices and abnormal RVFAC (<35%) had reasonable specificity for predicting negative outcomes, their sensitivity was low. The addition of strain or strain rate to abnormal RVFAC did not significantly improve the prediction of death or HT need. However, STE parameters showed better reproducibility than RVFAC.

Borrelli *et al*. [30] showed that the decline in longitudinal strain (Δ LS) between prior- and one month after Norwood operation was the strongest predictor of death or HT need (*p* = 0.01) in all patients irrespective of normal or abnormal RVFAC (Table 2). In this study, the reproducibility of STE was also better than RVFAC’s. One week before bidirectional cavopulmonary anastomosis (BCPA), Δ LS was significantly reduced in the event group compared to the rest of the HLHS patients. However, earlier Δ LS, one month after Norwood, was a better predictor than Δ LS one week before BCPA.

Additionally, Park *et al*. [31] examined the potential prognostic value of pre-TCPC STE in 135 patients with right or left dominant ventricle (Table 2). In this study, invasively measured pulmonary vascular resistance (PVR) and circumferential strain rate (CSR) were the only variables independently associated with a prolonged length of hospital stay (>14 days), with CSR showing better prognostic ability. Receiver operating characteristic curve analysis showed an area under the curve of 0.70 for circumferential strain rate and 0.64 for pulmonary vascular resistance, suggesting a potential role of STE during pre-TCPC assessment in addition to invasively assessed hemodynamic parameters.

Overall, the findings from these studies indicate that ventricular strain and strain rate, particularly STE-derived indices, may serve as potential prognostic markers in patients with single ventricle physiology.

Circumferential ventricular strain has been shown to have prognostic value in post-Fontan patients. In Ghelani *et al*.’s study [32] of 127 Fontan patients, low circumferential ventricular strain was found to have the strongest association with mortality or need for heart transplant (hazard ratio 1.3 per unit change, 95% confidence interval 1.1 to 1.5, *p* = 0.001) among echocardiographic variables (Table 2). The study also explored the prognostic role of cardiac magnetic resonance (CMR) imaging, with indexed ventricular end-diastolic volume being the stronger predictor of adverse outcomes among CMR parameters. When comparing echocardiography and CMR, neither technique was found to be superior in prognostic ability in this specific cohort. However, both techniques identified important factors for risk stratification.

## 4. The Role of STE in Atrial Function Assessment

The assessment of diastolic ventricular function can be improved by using atrial strain measured by STE, which can detect abnormalities before atrial volume changes are observed [33]. The atrial strain has three components: the reservoir strain, conduit strain, and active/pumping strain. In single ventricle patients, effective atrial function is crucial in ensuring adequate preload as diastolic ventricular filling depends more on atrial contraction [34].

Studies have shown that single ventricle patients exhibit increased atrial size and active atrial strain but decreased reservoir and conduit atrial strain compared to participants with biventricular hearts [35, 36]. Moreover, decreased atrial compliance and early diastolic emptying suggest the increased dependency of ventricular filling on atrial contraction. Lower right atrial strain (RAS) after initial palliation surgery has also been associated with a longer duration of milrinone therapy and a higher chance of digoxin use in single RV patients [37].

In Fontan patients, a decrease in global atrial strain has been observed, with no correlation between global atrial strain and invasively measured systemic ventricular filling pressure [38, 39]. However, atrial reservoir strain has been shown to correlate positively with cardiac index, and its decreased function is associated with adverse clinical outcomes, such as death, HT, arrhythmia, heart failure symptoms, protein-losing enteropathy, plastic bronchitis, seizures, and pleural effusions [39]. Atrial strain indices have also been shown to correlate with oxygen uptake during exercise, with higher atrial conduit strain, higher reservoir strain, and lower active/reservoir strain ratio correlating with higher VO2 [40]. However, more studies are needed to understand the role of atrial mechanics in the risk stratification of Fontan patients, as clinical outcomes result from several factors.

## 5. Discussion

Progressive decline in myocardial function and subsequent heart failure are among the complications which limit functional status and prognosis in patients with single ventricle physiology [41]. In these patients, heterogeneities in the cardiac morphology and the effects of loading conditions make a reliable assessment of myocardial function with conventional echocardiography difficult or even not feasible. On the other hand, speckle-tracking echocardiography does not depend on the geometry of the ventricles, is free of angle-dependency limitations and straightforward to employ [42, 43, 44, 45]. As a result, it is an appealing supplementary method for assessing single-ventricle patients.

There are several potential clinical implications for using STE imaging in the routine assessment of patients with single-ventricle physiology. Several echocardiography studies on single-ventricle patients have used STE indices to assess the longitudinal, radial and circumferential ventricular performance, mechanical dyssynchrony and atrial active, reservoir and conduit function [18, 20, 30, 31]. The results showed STE to be more sensitive than conventional echocardiography in detecting an early and progressive decline in the performance of the dominant ventricle [18, 20, 30, 31]. As such, serial interstage and post-Fontan completion STE assessment may help to early identify ventricular dysfunction and Fontan circulation failure and to allow prompt management of complications [18, 20, 22, 23, 26, 27]. Moreover, pre-and post-operative STE indices were associated with mortality, need for heart transplantation and prolonged hospitalization [13, 28, 29, 30, 31, 32]. Thus they serve as potential prognostic markers of adverse events and may play a role in optimizing risk stratification and informed patient counselling.

## 6. Conclusion

Despite the growing research in this field over the past years, most of the studies were single-center, retrospective, with small sample sizes of single ventricle patients. Even if STE is a handy and valuable tool for assessing single ventricle function, its definite diagnostic role must be proven through prospective, multi-center studies, including more patients with better representation of various single-ventricle anatomic types who will be followed up for a more extended period.
